# Long-term trajectory of cognitive performance in people with bipolar disorder and controls: 6-year longitudinal study

**DOI:** 10.1192/bjo.2021.66

**Published:** 2021-06-18

**Authors:** Timea Sparding, Erik Joas, Caitlin Clements, Carl M. Sellgren, Erik Pålsson, Mikael Landén

**Affiliations:** Department of Psychiatry and Neurochemistry, Institute of Neuroscience and Physiology, The Sahlgrenska Academy, University of Gothenburg, Sweden; Department of Psychiatry and Neurochemistry, Institute of Neuroscience and Physiology, The Sahlgrenska Academy, University of Gothenburg, Sweden; Psychology Department, University of Pennsylvania, USA; Department of Physiology and Pharmacology, Karolinska Institutet, Sweden; Department of Psychiatry and Neurochemistry, Institute of Neuroscience and Physiology, The Sahlgrenska Academy, University of Gothenburg, Sweden; Department of Psychiatry and Neurochemistry, Institute of Neuroscience and Physiology, The Sahlgrenska Academy, University of Gothenburg, Sweden; and Department of Medical Epidemiology and Biostatistics, Karolinska Institutet, Sweden

**Keywords:** Bipolar affective disorders, cognitive neuroscience, psychological testing, longitudinal, cognitive impairment

## Abstract

**Background:**

Cross-sectional studies have found impaired cognitive functioning in patients with bipolar disorder, but long-term longitudinal studies are scarce.

**Aims:**

The aims of this study were to examine the 6-year longitudinal course of cognitive functioning in patients with bipolar disorder and healthy controls. Subsets of patients were examined to investigate possible differences in cognitive trajectories.

**Method:**

Patients with bipolar I disorder (*n* = 44) or bipolar II disorder (*n* = 28) and healthy controls (*n* = 59) were tested with a comprehensive cognitive test battery at baseline and retested after 6 years. We conducted repeated measures ANCOVAs with group as a between-subject factor and tested the significance of group and time interaction.

**Results:**

By and large, the change in cognitive functioning between baseline and follow-up did not differ significantly between participants with bipolar disorder and healthy controls. Comparing subsets of patients, for example those with bipolar I and II disorder and those with and without manic episodes during follow-up, did not reveal subgroups more vulnerable to cognitive decline.

**Conclusions:**

Cognitive performance remained stable in patients with bipolar disorder over a 6-year period and evolved similarly to healthy controls. These findings argue against the notion of a general progressive decline in cognitive functioning in bipolar disorder.

## Background

Patients with bipolar disorder show cognitive impairment relative to healthy controls at the group level.^1–4^ However, the first systematic review that determined the prevalence of cognitive impairment in euthymic adults with bipolar disorder found large variation in the proportion of clinically relevant cognitive impairment across studies: the prevalence of impairment (5th per centile threshold) ranged from 5 to 58% depending on cognitive domain.^4^ Of note, all studies included in the review were cross-sectional.

A review by Cullen and colleagues^4^ noted that more severe or longstanding bipolar illness was associated with worse cognitive performance, which aligns with other studies where cognitive impairment has been associated with the number of manic episodes, admissions to hospital and use of antipsychotic medication.^5^ These findings suggest that cognitive performance might decline over the course of bipolar disorder. Indeed, a history of bipolar disorder increases the risk of dementia in older adults,^6^ and the risk increases as a function of the number of mood episodes in unipolar and bipolar affective disorders.^7^

Importantly, however, cross-sectional studies cannot demonstrate that mood episodes or illness duration cause cognitive impairment. Premorbid cognitive impairment might just as likely increase the risk of mood episodes. Most longitudinal studies of cognitive performance in bipolar disorder are short term, lack a control group, include only elderly patients, or used limited test batteries.^8^ To our knowledge, only three cohorts (reported in several publications) of patients with bipolar disorder and healthy controls have been followed for at least 5 years.^9–11^ Intriguingly, these studies suggest that the change in cognitive functioning over time does not differ between patients with bipolar disorder and healthy controls.^8,10,11^ In fact, only one subtest (verbal memory) in one study was found to decline more among patients than controls.^12^ In two studies, patients with bipolar disorder in fact improved on the delayed visual memory test^9^ and executive functioning.^13^

Cross-sectional studies have reported that subsets of patients with bipolar disorder feature clinically significant cognitive impairment whereas others perform within the normal range.^14–17^ It has been argued that overall group differences are driven by a subgroup of patients with marked levels of impairment.^4^ We have previously found that manic episodes predict decreased grey matter volume in dorsolateral prefrontal cortex at follow-up.^18^ We have also reported baseline findings from our study showing that although the majority of patients with bipolar disorder perform on a par with healthy controls, and patients with bipolar I and II disorder perform similarly,^3^ a subgroup (30%) showed memory impairments.^17^ It is not known whether this cognitive subgroup or bipolar subtypes show a different long-term cognitive trajectory.^8^

## Aims

The aims of this study were:

- to test if long-term changes in cognitive functioning in patients with bipolar disorder differ from normal human cognitive ageing, and
- to investigate if subsets of patients feature different cognitive trajectories.

To these ends, 72 patients with bipolar disorder and 59 healthy controls were tested with a comprehensive neuropsychological battery at baseline and then retested 6 years later.

## Method

### Participants

Data were collected within the framework of the St. Göran Bipolar Project, a naturalistic longitudinal prospective study.^3^ Patients were recruited from a bipolar disorder out-patient clinic at Northern Stockholm Psychiatry in Sweden. This clinic serves the northern Stockholm catchment area, which includes a spectrum of socioeconomic strata from wealthy areas with a high proportion of native-born Swedes to ethnically diverse areas with high deprivation indices. Patients in the catchment area who presented with symptoms of mania, hypomania or other signs of bipolar disorder were referred to this tertiary care bipolar out-patient unit for work-up and treatment. This means that effectively all new patients with bipolar disorder within the catchment area were referred for evaluation to this out-patient unit during the recruitment period.

Both new and existing eligible patients with ongoing treatment at the tertiary care bipolar out-patient unit were invited to participate in the study. Eligible patients were at least 18 years old, spoke a Scandinavian language and met criteria for bipolar I disorder, bipolar II disorder, bipolar disorder not otherwise specified (NOS), schizoaffective disorder bipolar type or cyclothymia according to DSM-IV criteria.

The Affective Disorder Evaluation (ADE) was used to establish the bipolar diagnoses.^19^ The ADE is a semi-structured interview developed for the Systematic Treatment Enhancement Program of Bipolar Disorder (STEP-BD).^19^ It includes the affective module of the Structural Clinical Interview for DSM-IV. The Mini International Neuropsychiatric Interview (MINI) was used in parallel to screen for comorbid psychiatric diagnoses.^20^ Board-certified psychiatrists or residents under psychiatric training completed the ADE and the MINI. A best estimate diagnosis was made at a case conference attended by experienced board-certified psychiatrists specialised in bipolar disorder. The final diagnostic assessment utilised all available sources of information, including the diagnostic interview, case records and information from next-of-kin when available.

All patients were mood stabilised at inclusion: most were euthymic while some presented with lingering subsyndromal symptoms (Table 1).^21^ Patients were remunerated for participation at follow-up but not at baseline.

**Table 1:** Demographic and clinical characteristics of individuals with bipolar disorder and healthy controls at baseline (*T*_1_) and follow-up (*T*_2_)

	Baseline (*T*_1_)			Follow-up (*T*_2_)		
	Bipolar disorder (*n* = 72)	Healthy controls (*n* = 59)	*P*	Bipolar disorder (*n* = 72)	Healthy controls (*n* = 59)	*P*
Age, years: mean (s.d.)	37.4 (12.1)	41.9 (14.6)	0.055^a^	–	–	–
Time elapsed between *T*_1_ and *T*_2_, years: median (IQR)	6.2 (5.7–7.1)	5.8 (5.5–6.0)	<0.001^a^	–	–	–
Gender, female, *n* (%)	44 (61)	29 (49)	0.170	–	–	–
Proportion with university studies, ≥3 years (%)	34(49)^b^	32(54)	0.576^c^	–	–	–
Occupational status – not working (%)	18(27)^d^	5(9)	0.011^c^	–	–	–
Premorbid IQ – WAIS-III: vocabulary – scaled score, mean (s.d.)	12 (3)	12 (3)	0.798^a^	–	–	–
Premorbid IQ – WAIS-III: vocabulary – raw score, mean (s.d.)	47.9 (9.5)	48.7 (9)	0.634^a^	–	–	–
WAIS-III: Full Scale IQ, mean (s.d.)	108 (14)	117 (11)	<0.001^a^	–	–	–
Age at onset, years: mean (s.d.)	19 (10)	–	–	–	–	–
YMRS, mean (s.d.)	1.8 (0.2)	0 (0.00)	<0.001^a^	0.8 (0.2)	0.5 (1.2)	0.24^a^
MADRS, mean (s.d.)	4.7 (6)	0 (0.00)	<0.001^a^	3.4 (4)	1.7 (3)	0.006^a^
GAF Function, mean (s.d.)	69 (11)	79 (6)	<0.001^a^	65 (10)	82 (7)	<0.001^a^
GAF Symptom, mean (s.d.)	69 (11)	79 (6)	<0.001^a^	66 (9)	81 (7)	<0.001^a^
Lithium, *n* (%)	45 (63)	–	–	45 (63)	–	–
Mood stabilisers (excluding lithium), *n* (%)	19 (26)	–	–	18 (25)	–	–
Antipsychotics, *n* (%)	14 (19)	–	–	20 (28)	–	–
Antidepressants, *n* (%)	31 (43)	–	–	31 (43)	–	–
Number of depressive episodes, *T*_1_ – *T*_2_, mean (s.d.)	–	–	–	3.3 (7.3)	–	–
Number of mixed episodes, *T*_1_ – *T*_2_, mean (s.d.)	–	–	–	0.8 (2.5)	–	–
Number of hypomanic episodes, *T*_1_ – *T*_2_, mean (s.d.)	–	–	–	1.9 (8.6)	–	–
Number of manic episodes, *T*_1_ – *T*_2_, mean (s.d.)	–	–	–	1.4 (6.2)	–	–
Bipolar disorder I with any mixed or/and manic episode between *T*_1_ – *T*_2_, *n* (%)	–	–	–	23 (52)^e^	–	–
Bipolar disorder I and II with any depressive episode between *T*_1_ – *T*_2_, *n* (%)	–	–	–	51 (73)^f^	–	–

IQR, interquartile range; WAIS, Wechsler Adult Intelligence Scale – version III; YMRS, Young–Ziegler Mania Rating Scale; MADRS, Montgomery–Åsberg Depression Rating Scale; GAF, Global Assessment of Functioning;

a. Independent *t*-test.

b. Total *n* = 69.

c. Pearson χ².

d. Total *n*= 68.

e. Total *n*=44.

f. Total *n*=70.

Statistics Sweden randomly selected and contacted population-based controls from the same catchment area by mail. Eligible controls who volunteered to participate were scheduled for an interview and testing. A psychiatrist used the MINI and selected parts of the ADE to screen for psychiatric disorders. Exclusion criteria for controls were any current psychiatric disorder, any neurological condition other than mild migraine, drug or alcohol use disorders (based on the Alcohol Use Disorder Identification Test, Drug Use Disorder Identification Test and serum levels of carbohydrate-deficient transferrin), untreated endocrinological disorders, pregnancy and a first-degree relative with bipolar disorder or schizophrenia. Controls were remunerated for participation at both baseline and follow-up. Details of the recruitment of both patients and controls can be found elsewhere.^22^

All study participants provided oral and written informed consent to participate in the study, which was approved by the Stockholm Regional Ethical Review Board.

The diagnostic procedure was the same for all patients and the ADE was used to establish the bipolar diagnoses. For the present study, we selected the subset of study participants included in the St. Göran study diagnosed with bipolar disorder type I or II. Inclusion criteria for participants with bipolar disorder were

- meeting DSM-IV criteria^23^ for bipolar disorder type I or type II at baseline;
- stable mood at the cognitive assessments as judged by the treating physician (i.e. not suffering from an acute mood episode), and
- completion of cognitive assessment at baseline and follow-up.

Inclusion criteria for controls was completion of cognitive testing at both baseline and follow-up. This yielded 44 patients with bipolar I disorder, 28 patients with bipolar II disorder and 59 healthy controls. There were no participants with intellectual disability included in the current study.

Patients with common comorbid diagnoses (such as attention-deficit hyperactivity disorder, anxiety disorder or borderline personality disorder) were not excluded given our aim to study the natural course of cognitive functioning in a representative clinical sample of patients with bipolar disorder.

We dichotomised the patient group along three factors to investigate potential differences in cognitive trajectories:

- bipolar I disorder (*n* = 44) versus bipolar II disorder (*n* = 28),
- bipolar I disorder with any manic and/or mixed episode between baseline and follow-up (*n* = 23) versus bipolar I disorder with no manic or mixed episodes between baseline and follow-up (*n* = 21),
- cognitive impairment at baseline (*n* = 17) as defined in a previous study^17^ versus no cognitive impairment at baseline (*n* = 43).

### Clinical measures

Information on educational attainment, occupational status, medication, age of first psychiatric symptoms, number of affective episodes and lifetime history of psychosis was recorded at baseline. Severity of illness was rated using the Clinical Global Impression (CGI) rating scale.^24^ Overall psychological, social, and occupational functioning was assessed with the Global Assessment of Functioning (GAF) scale.^23^ Current depressive and manic symptoms were evaluated with the Montgomery–Åsberg Depression Rating Scale (MADRS)^25^ and the Young–Ziegler Mania Rating Scale (YMRS).^26^

The assessments were repeated at follow-up, and the number of mood episodes since baseline were recorded. Patients completed baseline diagnostic and cognitive assessments on different days because of the duration of the assessments. All follow-up assessments and control baseline assessments were completed on one day.

### Cognitive test procedure

Study participants completed a comprehensive cognitive test battery at baseline and at follow-up 5–7 years later. The mean time elapsed between baseline and follow-up was somewhat shorter for healthy controls (5.83 years) than for patients (6.23 years). Patients were in a stable mood at time of the cognitive assessment. Mood symptoms were rated using MADRS and YMRS. No patient scored above 11 points on the YMRS at baseline or follow-up. With respect to MADRS, six patients at baseline and one patient at follow-up scored >14 points.

### Cognitive testing at baseline

A licensed psychologist tested patients’ cognitive functioning over two sessions. The controls were assessed by trained research associates, supervised by a licensed psychologist, at a single session.

Five stand-alone tests from the Delis–Kaplan Executive Function System (D-KEFS)^27^ were used: Color-Word Interference Test (CWIT), Design Fluency Test (DFT), Tower Test, Trail Making Test (TMT) and Verbal Fluency Test (VFT); together with all the tests from the Wechsler Adult Intelligence Scale – version III (WAIS-III)^28^ except the Letter–Number Sequencing, Comprehension, and Object Assembly. The battery also included the Continuous Performance Test II (CPT-II), the Rey Complex Figure Test (RCFT), and the Claeson–Dahl Verbal Learning and Retention Test.^29^ The latter is a word list learning task that presents ten words for a maximum of ten learning trials. The combined cognitive battery thus covers a broad range of cognitive abilities including attentional capacity, processing, working memory/mental tracking, concentration/focused attention, verbal memory, visual memory, verbal functions/language, construction and motor performance, concept formation/reasoning, planning and decision-making, and self-regulation/self-monitoring. The participants’ baseline performance on cognitive tests has been published previously.^2,3,17^

### Cognitive testing at follow-up

At follow-up, both patients and controls were tested during a single session by trained research associates who were supervised by a licensed psychologist. The time required to complete the cognitive testing was approximately 4 h. The same test battery was administered at baseline and follow-up, with the exception of two tests from D-KEFS that were omitted at follow-up due to time constraints: the DFT and the Tower Test.

The following cognitive subtests were used for the present study.

- From D-KEFS: CWIT condition 3 (inhibition) and condition 4 (inhibition/switching); VFT category fluency and switching; and TMT condition 4 (switching).
- RCFT copy and immediate recall.
- From WAIS-III: vocabulary (used as proxy for premorbid cognitive ability); Similarities, Block Design, Digit symbol substitution test; Symbol Search; and Digit-Symbol-Coding-Incidental Learning; Pairing.
- Claeson-Dahl Verbal Learning Test.
- CPT-II: Omissions.

### Statistical procedures

Group differences between patients with bipolar disorder and healthy controls regarding demographic and clinical characteristics at baseline and follow-up were analysed with independent *t*-tests and Pearson χ²-tests.

The main analysis tested if the change in cognitive function over time differed between patients and controls. To this end, we conducted repeated measures ANCOVAs with group (patients versus healthy control) as a between-subject factor and with age as a covariate to correct for individual differences in age at baseline in the main analyses. In this procedure, the significance of group and time interaction was investigated. This procedure was repeated on the raw scores on the 14 cognitive tests measuring different cognitive domains. We performed a Bonferroni correction to correct for multiple testing. With 14 tests in the main analysis, the alpha level was set to 0.0036 (0.05/14).

We then conducted subgroup analyses in the same way as the main analyses with age at baseline as a covariate. First, potential differences in the cognitive change over time between bipolar I disorder and bipolar II disorder subgroups were investigated with repeated measures ANCOVAs with group (bipolar I disorder and bipolar II disorder) as a between-subjects factor. Second, we compared the subgroup of patients that were cognitively impaired at baseline with the rest of patients with bipolar disorder using repeated measures ANCOVAs with group as a between-subjects factor. The cognitively impaired subgroup was identified and defined by baseline scores in an earlier study of the same cohort.^17^ Third, the potential influence of manic or mixed episodes during the follow-up period was assessed by a repeated measures ANCOVAs with group (any manic or mixed episodes, and no manic or mixed episodes) as a between-subjects factor.

Finally, we compared participants who participated in follow-up with those who did not. Pearson χ²-tests were used to investigate potential differences in gender or educational level. Independent-samples *t*-tests were used to compare baseline intelligence and cognitive ability.

## Results

At baseline, 127 patients with bipolar I disorder or bipolar II disorder were assessed with the cognitive test battery, of these 72 were available for retesting at follow-up. A total of 113 healthy controls were enrolled at baseline, and of these 59 were available for retesting at follow-up. The main reasons for attrition among patients were that the individuals did not wish to participate (*n* = 18), moved out of the area (*n* = 3), died (*n* = 4), various other reasons (*n* = 9) or lost to follow-up (*n* = 21). The main reasons for attrition among healthy controls were that the individuals did not wish to participate (*n* = 17), could not be reached (*n* = 7), pregnancy (*n* = 1), newly diagnosed multiple sclerosis (*n* = 1) or lost to follow-up (*n* = 28). Those who undertook and those who did not undertake the follow-up were compared with respect to baseline characteristics. Neither gender nor educational level differed between completers and non-completers. In patients, there was no difference in age or IQ between completers and non-completers. However, controls who completed follow-up had significantly higher IQ (mean 117 (s.d. = 11) *v.* 110 (s.d. = 10); *t*(106) = −3,76, *P* < 0.001) and were older (mean 42 (s.d. = 15) *v.* 34 (s.d. = 11) years; *t*(111) = −3.11, *P* = 0.002) than controls who did not participate in the follow-up.

Table 1 displays demographic and clinical variables of patients with bipolar disorder and healthy controls for baseline (*T*_1_) and follow-up (*T*_2_).^21^ The groups did not differ with regard to age, gender, premorbid intellectual ability (WAIS-III vocabulary) or proportion with university studies. However, there was a difference in Full Scale IQ between patients with bipolar disorder and healthy controls.

### Long-term changes in cognition

To investigate if long-term change in cognitive functioning in patients with bipolar disorder differs from normal human ageing, we compared the interaction effect of ‘group × time' in patients with bipolar disorder with healthy controls for each cognitive test. Table 2 shows each groups’ performance on cognitive tests at baseline (*T*_1_) and follow-up (*T*_2_) and statistics for the ‘group × time' interaction, adjusted for age at baseline. The changes in cognitive functioning over time did not differ between patients and healthy controls.

**Table 2:** Individuals with bipolar disorder in comparison with healthy controls with respect to the change in performance on cognitive tests between baseline (*T*_1_) and follow-up (*T*_2_)^a^

	Comparison of long-term trajectory of cognition in bipolar disorder and healthy controls								
	Bipolar disorder			Healthy controls			Group × time^b^		
Cognitive test	*T*_1_ mean (s.d.)	*T*_2_ mean (s.d.)	*n*	*T*_1_ mean (s.d.)	*T*_2_ mean (s.d.)	*n*	*F*	*P*	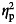
Color Word Interference Test 3: Inhibition	53 (13)	57 (25)	61	47 (9)	47(9)	57	4.09	0.045	0.034
Color Word Interference Test 4: Inhibition/Switching	61 (15)	62 (21)	61	55 (12)	55(17)	56	0.056	0.81	0.00
Verbal Fluency Test: Category Fluency	49 (13)	50 (11)	66	54 (9)	55 (11)	55	0.046	0.83	0.000
Verbal Fluency Test: Switching	15 (3)	16 (4)	65	17 (3)	16 (2)	55	3.181	0.08	0.001
Trail Making Test 4: Switching	78 (29)	78 (35)	56	62 (17)	65 (27)	55	0.16	0.69	0.003
Rey Complex Figure test: time to copy	198 (103)	190 (97)	66	152 (59)	156 (71)	55	0.71	0.40	0.006
Rey Complex Figure test: immediate recall	19 (7)	18 (8)	67	22 (6)	22 (6)	57	1.946	0.17	0.016
WAIS-III: Similarities	23 (6)	25 (6)	70	27 (4)	27 (5)	58	5.96	0.02	0.045
WAIS-III: Block design	46 (10)	47 (14)	71	51 (10)	52 (10)	59	0.00	0.99	0.000
WAIS-III: Digit symbol substitution test	73 (18)	68 (19)	71	78 (16)	73 (17)	58	0.001	0.97	0.000
WAIS-III: Symbol Search	33 (9)	33 (10)	71	36 (8)	38 (8)	59	2.14	0.15	0.017
WAIS-III - Digit-Symbol-Coding-Incidental Learning	13 (5)	12 (5)	54	15 (4)	14 (5)	41	0.66	0.42	0.007
Claeson-Dahl Verbal Learning (and Retention) Test	87 (71)	77 (62)	34	64 (56)	49 (48)	50	0.2	0.66	0.002
Conners’ Continuous Performance Test II Omissions.	5 (15)	5 (9)	45	2 (3)	2 (3)	42	0.073	0.79	0.001

WAIS-III, Wechsler Adult Intelligence Scale III.

a. Results are presented as mean raw scores with standard deviation (s.d.) and statistics for the group × time interaction, adjusted for age at baseline.

b. Adjusted for age at baseline, 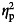 = partial eta squared = effect size.

### Subgroup analyses

To investigate if long-term change in cognitive functioning differs between subgroups of bipolar disorder, we tested the interaction effect of ‘group × time' in three subgroup analyses (Supplementary Tables 1–3 available at https://doi.org/10.1192/bjo.2021.66):

- bipolar I disorder versus bipolar II disorder;
- the cognitively impaired subgroup identified at baseline versus the remainder of patients; and
- patients with a manic or mixed episode during the follow-up versus those without such episodes.

Supplementary Table 1 shows that the diagnostic subgroups bipolar I disorder and bipolar II disorder did not differ regarding change in cognition over the study period. Supplementary Table 2 shows that the cognitively impaired subgroup of individuals with bipolar disorder identified at baseline remained stable and did not change more or less than patients with bipolar disorder with normal performance at baseline. Finally, Supplementary Table 3 shows that patients who had at least one manic or mixed episode did not show greater cognitive decline than those with no manic or mixed episode.

## Discussion

### Main findings

We compared the trajectory of cognitive performance in 72 patients with bipolar disorder with 59 healthy controls over a period of 6 years. We used a comprehensive cognitive test battery tapping into important aspects of cognitive functioning including processing speed, different aspects of memory and set-shifting. The main finding is that patients with bipolar disorder did not differ from healthy individuals of similar age and education with respect to the change in cognitive performance over the 6-year time period.

Drilling deeper into patient subgroups, we found no difference between bipolar I and II disorder regarding change in cognition over the study period. Considering that patients with cognitive impairment might be at higher risk for further deterioration, we specifically followed the subset of patients that were cognitively impaired at baseline,^17^ but found that this group remained cognitively stable as well. Finally, patients who had at least one manic or mixed episode during the 6-year follow-up did not show greater cognitive decline than those with no manic or mixed episode during follow-up. Taken together, we find no evidence to suggest that patients with bipolar disorder are at higher risk for cognitive decline than healthy controls.

### Interpretation of our findings and comparison with other studies

Cognitive ageing is a complex process that differs across individuals and cognitive domains.^30^ Certain cognitive functions show little age-associated decline, for example verbal ability, some numerical abilities and general knowledge. Other abilities decline from middle age and onwards, for example memory, executive functions, processing speed and reasoning. It is therefore necessary to make comparisons with a healthy control group when investigating if patients with bipolar disorder show pathological cognitive decline. The number of previous long-term studies that include a control group is, however, limited.^8^

We identified three cohorts (reported in seven publications) with long-term follow-up (≥5 years) of cognition in patients with bipolar disorder in the same age range as the current study: Mora and colleagues^11^ followed a cohort with 28 patients and 26 healthy controls over 6 years. They found that cognitive functioning remained stable on average. A second cohort of patients with bipolar I disorder and healthy controls have been reported on in four publications: first, Santos and colleagues^12^ found unchanged cognitive functioning over 5 years in their study of 62 patients and 40 healthy controls – except for a progressive decline in delayed verbal recall in patients – and no association with clinical or treatment variables, or clinical course during the follow-up period.

Second, Sánchez-Morla^10^ found stable cognitive performance in 76 patients with bipolar disorder and 40 controls, the majority of whom had been accounted for by Santos and colleagues.^12^ Third, Jiménez-López^31^ investigated both cognitive functioning and functional outcomes in patients with a history of psychotic symptoms (*n* = 44) with patients with bipolar disorder without such symptoms (*n* = 34) and found no evidence of progression in any of the groups. Finally, López-Villarreal^13^ examined the same cohort, and also found stable cognitive performance except for executive functioning that was slightly improved. They concluded that the best predictor for psychosocial functioning was course of illness during the follow-up period.

A third cohort has been examined by Ryan and colleagues^32^ who specifically studied executive functioning in a sample of predominately patients with bipolar I disorder (*n* = 91). They found no difference compared with 17 healthy controls. In a different approach in the same cohort, Hinrichs and colleagues^9^ investigated the influence of cognitive reserve factors (such as education and IQ) in 159 patients with bipolar disorder (bipolar I disorder, bipolar II disorder and bipolar disorder NOS) and 54 healthy controls. They found that change in neurocognitive performance over 5 years did not differ between the groups with one exception: patients with bipolar disorder slightly improved in delayed visual memory.

A possible explanation for the absence of cognitive decline in this study is that cognition worsens in older ages (for example above 60 years of age) and that our study cohort is too young (mean age 37) to capture this. Speaking against this notion, however, several previous studies that included elderly populations also failed to demonstrate faster decline in cognitive functioning in people with bipolar disorder compared with health controls.^33–35^

Our findings thus align with previous studies and combined evidence strongly suggests that there is no progressive cognitive decline in bipolar disorder compared with healthy controls during a 5–6 years’ time span.

Susceptibility to cognitive decline might, however, differ across clinical subtypes of bipolar disorder (i.e. type I and type II). Few studies have controlled for bipolar disorder subtype^8^ but a recent meta-analysis concluded that people with bipolar I disorder performed significantly worse than those with bipolar II disorder with respect to global cognition, verbal memory, processing speed, as well as executive functioning speed and accuracy.^36^ The present study is the first to investigate cognitive change over time in people with bipolar I disorder relative to those with bipolar II disorder. We neither found cognitive differences at baseline^3^ nor differences in long-term cognitive change between bipolar I disorder and bipolar II disorder in the present study.

Bora & Özerdem^8^ stress the importance of monitoring cognitive decline in patients with frequent manic episodes, a notion based on findings in cross-sectional studies.^36,37^ Only a single longitudinal study has linked a higher number of manic and hypomanic episodes during follow-up to a greater decrease in neurocognitive composite index.^10^ We nevertheless expected greater cognitive decline in patients with manic episode(s) as we found that manic episodes predicted decreased grey matter volume in dorsolateral prefrontal cortex at follow-up.^18^ We were thus surprised to find no cognitive differences between patients with and without manic episode(s) during the 6-year follow-up.

Our findings add to other longitudinal studies that have failed to link cognitive decline to manic episodes. In fact, longitudinal studies have not been able to link any clinical feature to the course of cognitive functioning,^12,31^ except for a correlation between a single test (reaction time of CPT-II) and illness duration,^11^ and the one association with manic and hypomanic episodes mentioned above.^10^

Several studies have defined cognitive subtypes^14–16,38,39^ to explain the significant heterogeneity of cognitive functioning in bipolar disorder.^4^ In this vein, we identified a subgroup of patients with bipolar disorder at baseline assessment that showed significantly lower overall cognitive performance and pronounced impairments in verbal and visual memory.^17^ We hypothesised that this subgroup might be more vulnerable to further cognitive deterioration. However, in the current study we find that this subgroup's cognitive impairment remains remarkably stable over time.

### Strengths and limitations

This study followed a clinical cohort of patients with bipolar disorder for over 6 years, along with population-based controls recruited from the same catchment area. As effectively all new patients with bipolar disorder within the catchment area were referred for evaluation to our out-patient unit during the recruitment period, the sample is representative of the bipolar disorder population receiving psychiatric care in a metropolitan area. The same rigorous neuropsychological test battery capturing key cognitive functions was administered at baseline and follow-up.

The first limitation to consider is attrition bias, which is expected because of the long follow-up period. We were able to retest 57% of patients and 52% of controls. Retention rates did not differ significantly by group. There were no baseline differences between those participants who dropped out and those who completed the study in the patient group with respect to baseline characteristics. It cannot be ruled out, however, that individuals who dropped out might have had a different illness course or been less functionally stable during the follow-up time. In the control group, completers were older and had higher IQ than those who dropped out. These differences are, however, unlikely to have biased the results since the patient and control completer groups did not significantly differ in age or verbal IQ.

Second, we only assessed cognitive function at two time points but several measuring points might be needed to further assess stability and determinants of cognitive functioning. The benefits with repeated cognitive testing must, however, be weighed against the risk of practice effects. Third, although our sample size is on par or larger than previous studies, even larger samples might be needed to capture subtle differences between cases and controls. A larger sample size is warranted to take into account the heterogeneity in cognition, illness course and comorbidities that are seen in bipolar disorder. Further, our results suggest that effect sizes between groups on all cognitive measure are of small or medium magnitude. The study might nevertheless be underpowered to detect signals in the subgroup analyses, particularly given the limited sample of patients with bipolar I disorder with and without manic episodes.

Fourth, we did not correct for multiple testing. However, our study was negative meaning that correcting for multiple testing would not change the results. Fifth, in Sweden approximately 40% of the adult population has at least 2 years of tertiary education. Among patients in the present study, 49% had ≥3 years of university studies. It has previously been suggested that many studies have a sampling bias favouring patients who are cognitively impaired,^40^ which thus does not seem to be the case in the present study. Finally, we studied middle-aged individuals. According to the theory of accelerating ageing, it is possible that group differences in cognitive functioning may emerge at an older age.

In summary, the current study found that cognitive functioning in patients with bipolar disorder over a 6-year period is comparable with normal human ageing. No subgroups of patients emerged as more susceptible to cognitive decline than other groups.

## Data Availability

The data that support the findings of this study are available on reasonable request from the corresponding author, T.S. The data are not publicly available because of Swedish legal restrictions.

## Supplementary material

For supplementary material accompanying this paper visit http://dx.doi.org/10.1192/bjo.2021.66. click here to view supplementary material
