# Supplementary material for: Long-term trajectory of cognitive performance in people with bipolar disorder and controls: 6-year longitudinal study
Source: BJPsych Open. 2021 Jun 18;7(4):e115. doi: 10.1192/bjo.2021.66 (PMC8240122; doi:10.1192/bjo.2021.66)
Supplement: Supplementary file 1 [file S2056472421000661sup001.docx]

Supplementary

| **Supplementary Table 1**  Patients with bipolar disorder type I are compared to type II with respect to the change in performance on cognitive tests between baseline (T1) and follow‑up (T2). Results are presented as *M*(SD) raw scores and statistics for the group x time interaction, adjusted for age at baseline. | | | | | | | | | | |  |  |  |
| --- | --- | --- | --- | --- | --- | --- | --- | --- | --- | --- | --- | --- | --- |
|  | Comparison of long-term trajectory of cognition in individuals with BD type I and II | | | | | | | | | | | |  |
|  | Bipolar I disorder | | | Bipolar II disorder | |  | Group x time^a^ | | | | | |  |
| Cognitive test | T1 | T2 | N | T1 | T2 | N | F | *p* | η_p_^2^ | | | |  |
| CWIT 3: Inhibition | 52(14) | 58(30) | 37 | 54(12) | 56(15) | 24 | 0.46 | 0.5 | 0.008 | | | |  |
| CWIT 4: Inhibition/Switching | 62(17) | 64(24) | 37 | 60(13) | 59(15) | 24 | 0.36 | 0.55 | 0.006 | | | |  |
| VFT: CF | 49(12) | 50(12) | 42 | 48(13) | 49(11) | 24 | 0.005 | 0.94 | 0.000 | | | |  |
| VFT: Switching | 15(3) | 15(4) | 41 | 15(3) | 16(4) | 24 | 0.04 | 0.84 | 0.001 | | | |  |
| TMT 4: Switching | 81(33) | 80(39) | 34 | 73(22) | 76(30) | 22 | 0.31 | 0.58 | 0.006 | | | |  |
| RCFT: time to copy | 205(114) | 185(80) | 39 | 189(84) | 197(119) | 27 | 1.22 | 0.27 | 0.019 | | | |  |
| RCFT: IR | 19(7) | 18(8) | 39 | 20(8) | 17(8) | 28 | 1.09 | 0.30 | 0.017 | | | |  |
| WAIS-III: Similarities | 24(5) | 25(5) | 42 | 23(6) | 26(7) | 28 | 2.13 | 0.15 | 0.031 | | | |  |
| WAIS-III:  Block design | 45(13) | 45(13) | 43 | 47(15) | 49(15) | 23 | 0.31 | 0.58 | 0.005 | | | |  |
| WAIS-III: DSST | 72(17) | 67(20) | 43 | 73(19) | 68(18) | 28 | 0.01 | 0.91 | 0.000 | | | |  |
| WAIS-III: SS | 34(8) | 33(9) | 43 | 33(9) | 33(10) | 28 | 0.12 | 0.73 | 0.002 | | | |  |
| WAIS-III - DSC IL: Pairing | 14(4) | 13(5) | 32 | 12(7) | 11(6) | 22 | 0.09 | 0.76 | 0.002 | | | |  |
| CD VL | 80(71) | 75(65) | 19 | 96(72) | 81(59) | 15 | 0.40 | 0.53 | 0.012 | | | |  |
| CPT-II: Omissions | 7(20) | 7 (11) | 25 | 3(3) | 4(4) | 20 | 0.08 | 0.77 | 0.002 | | | |  |
| *Note.* ^a^ adjusted for age at baseline, η_p_^2^ = partial eta squared= effect size, abbreviations cognitive tests: CWIT=Color Word Interference Test, VFT=Verbal Fluency Test, CF=Category Fluency, TMT=Trail Making Test, RCFT=Rey Complex Figure test, IR=Immediate Recall, WAIS-III=Wechsler Adult Intelligence Scale III, DSST=Digit symbol substitution test, SS=Symbol Search, DSC IL=Digit-Symbol-Coding-Incidental Learning, CD VLT=Claeson-Dahl Verbal Learning (and Retention) Test, CPT-II: Omissions= Conners' Continuous Performance Test II Omissions. | | | | | | | | | | |  |  |  |
|  |  |  |  |  |  |  |  |  |  |  | | | |

| **Supplementary Table 2**  The cognitive subtype group of bipolar disorder patients identified at baseline were compared with the rest of the bipolar disorder patients with respect to the change in performance on cognitive tests between baseline (T1) and follow‑up (T2). Results are presented as mean (SD) raw scores and statistics for the group x time interaction, adjusted for age at baseline. | | | | | | | | | |
| --- | --- | --- | --- | --- | --- | --- | --- | --- | --- |
|  | Comparison of long-term trajectory of cognition in bipolar cognitive subtype group and other bipolar disorder patients | | | | | | | | |
| Cognitive test | Cognitive subtype group | | | Bipolar disorder patients | | | Group x time^a^ | | |
|  | T1 | T2 | N | T1 | T2 | N | F | *p* | η_p_^2^ |
| CWIT 3: Inhibition | 53(10) | 53(12) | 13 | 54(14) | 60 30) | 38 | 0.97 | 0.33 | 0.020 |
| CWIT 4: Inhibition/Switching | 6718) | 63(14) | 13 | 59(13) | 61(24) | 38 | 1.17 | 0.28 | 0.024 |
| VFT: CF | 43(12) | 44(9) | 15 | 49(13) | 50(11) | 40 | 0.52 | 0.82 | 0.001 |
| VFT: Switching | 13(3) | 14(3) | 15 | 15(3) | 16(4) | 39 | 2.51 | 0.12 | 0.047 |
| TMT 4: Switching | 97(34) | 99(47) | 13 | 74(25) | 72(29) | 38 | 0.16 | 0.90 | 0.000 |
| RCFT: time to copy | 257(130) | 243(152) | 16 | 169(77) | 172(63) | 40 | 0.13 | 0.72 | 0.002 |
| RCFT: IR | 15(6) | 16(8) | 16 | 21(7) | 19(8) | 40 | 2.25 | 0.14 | 0.041 |
| WAIS-III: Similarities | 23(5) | 25(3) | 17 | 24(5) | 26(6) | 42 | 0.74 | 0.39 | 0.013 |
| WAIS-III:  Block design | 41(12) | 38(13) | 17 | 49(12) | 51(13) | 42 | 1.67 | 0.20 | 0.029 |
| WAIS-III: DSST | 65(17) | 56(18) | 17 | 75(17) | 70(18) | 42 | 0.9 | 0.35 | 0.016 |
| WAIS-III: SS | 29(8) | 28(9) | 17 | 35(8) | 35(99) | 42 | 0.41 | 0.53 | 0.007 |
| WAIS-III - DSC IL: Pairing | 12(4) | 11(4) | 14 | 14(5) | 12(12) | 31 | 0.81 | 0.78 | 0.002 |
| CD VL | 101(74) | 90(80) | 5 | 80(72) | 74(62) | 24 | 0.023 | 0.88 | 0.001 |
| CPT-II: Omissions | 15(35) | 9(12) | 8 | 3(3) | 3(8) | 31 | 1.46 | 0.24 | 0.039 |
| *Note.* ^a^ adjusted for age at baseline, η_p_^2^ = partial eta squared= effect size, abbreviations cognitive tests: CWIT=Color Word Interference Test, VFT=Verbal Fluency Test, CF=Category Fluency, TMT=Trail Making Test, RCFT=Rey Complex Figure test, IR=Immediate Recall, WAIS-III=Wechsler Adult Intelligence Scale III, DSST=Digit symbol substitution test, SS=Symbol Search, DSC IL=Digit-Symbol-Coding-Incidental Learning, CD VLT=Claeson-Dahl Verbal Learning (and Retention) Test, CPT-II: Omissions= Conners' Continuous Performance Test II Omissions. | | | | | | | | | |

| **Supplementary Table 3**  Individuals with BD I who suffered from manic or/and mixed episodes between baseline and follow‑up were compared to patients with BD I with no manic or/and mixed episodes during follow-up. Results are presented as *M*(SD) raw scores and statistics for the group x time interaction, adjusted for age at baseline. | | | | | | | | | |
| --- | --- | --- | --- | --- | --- | --- | --- | --- | --- |
|  | Comparison of long-term trajectory of cognition in individuals with BD and manic or/and mixed episodes between baseline and follow and those without such episodes | | | | | | | | |
| Cognitive test | BDI with **any** manic or/and mixed episodes during follow-up | | | BD I with **no** manic or/and mixed episode during follow-up | | | Group x time^a^ | | |
|  | T1 | T2 | N | T1 | T2 | N | F | *p* | η_p_^2^ |
| CWIT 3: Inhibition | 51(13) | 61(38) | 19 | 54(15) | 55(19) | 18 | 1.44 | 0.24 | 0.040 |
| CWIT 4: Inhibition/Switching | 62(17) | 64(27) | 19 | 62(17) | 64(20) | 18 | 0.028 | 0.87 | 0.001 |
| VFT: CF | 50(13) | 51(12) | 21 | 48(11) | 49(13) | 21 | 0.13 | 0.72 | 0.003 |
| VFT: Switching | 15(3) | 15(4) | 21 | 15(4) | 15(15) | 20 | 0.257 | 0.62 | 0.007 |
| TMT 4: Switching | 75(23) | 67(18) | 18 | 88(41) | 94(51) | 16 | 1.28 | 0.27 | 0.040 |
| RCFT: time to copy | 179(91) | 189(74) | 22 | 238(135) | 179(90) | 17 | 3.35 | 0.075 | 0.085 |
| RCFT: IR | 20(6) | 19(8) | 22 | 17(8) | 16(7) | 17 | 0.005 | 0.95 | 0.000 |
| WAIS-III: Similarities | 23(5) | 24(5) | 22 | 25(5) | 36(5) | 20 | 0.008 | 0.93 | 0.000 |
| WAIS-III:  Block design | 47(13) | 47(14) | 22 | 44(12) | 44(13) | 21 | 0.024 | 0.88 | 0.001 |
| WAIS-III: DSST | 74(17) | 69(19) | 21 | 71(16) | 66(21) | 21 | 0.009 | 0.92 | 0.000 |
| WAIS-III: SS | 33(9) | 33(10) | 22 | 33(9) | 33(8) | 21 | 0.34 | 0.53 | 0.010 |
| WAIS-III - DSC IL: Pairing | 13(4) | 12(5) | 17 | 14(5) | 13(5) | 15 | 0.017 | 0.9 | 0.001 |
| CD VL | 65(60) | 71(65) | 8 | 91(79) | 78(69) | 11 | 0.44 | 0.52 | 0.027 |
| CPT-II: Omissions | 3(3) | 7(12) | 12 | 10(28) | 6(10) | 13 | 0.69 | 0.42 | 0.030 |
| *Note.* ^a^ adjusted for age at baseline, η_p_^2^ = partial eta squared= effect size, abbreviations cognitive tests: CWIT=Color Word Interference Test, VFT=Verbal Fluency Test, CF=Category Fluency, TMT=Trail Making Test, RCFT=Rey Complex Figure test, IR=Immediate Recall, WAIS-III=Wechsler Adult Intelligence Scale III, DSST=Digit symbol substitution test, SS=Symbol Search, DSC IL=Digit-Symbol-Coding-Incidental Learning, CD VLT=Claeson-Dahl Verbal Learning (and Retention) Test, CPT-II: Omissions= Conners' Continuous Performance Test II Omissions | | | | | | | | | |
